# Transcriptional Control of Mitosis: Deregulation and Cancer

**DOI:** 10.3389/fendo.2015.00060

**Published:** 2015-05-05

**Authors:** Somsubhra Nath, Dishari Ghatak, Pijush Das, Susanta Roychoudhury

**Affiliations:** ^1^Cancer Biology and Inflammatory Disorder Division, CSIR-Indian Institute of Chemical Biology, Kolkata, India

**Keywords:** mitosis, aneuploidy, cancer, transcription, mutation

## Abstract

Research over the past few decades has well established the molecular functioning of mitosis. Deregulation of these functions has also been attributed to the generation of aneuploidy in different tumor types. Numerous studies have given insight into the regulation of mitosis by cell cycle specific proteins. Optimum abundance of these proteins is pivotal to timely execution of mitosis. Aberrant expressions of these mitotic proteins have been reported in different cancer types. Several post-transcriptional mechanisms and their interplay have subsequently been identified that control the level of mitotic proteins. However, to date, infrequent incidences of cancer-associated mutations have been reported for the genes expressing these proteins. Therefore, altered expression of these mitotic regulators in tumor samples can largely be attributed to transcriptional deregulation. This review discusses the biology of transcriptional control for mitosis and evaluates its role in the generation of aneuploidy and tumorigenesis.

## Introduction

The propagation of eukaryotic life is orchestrated by the generation of descendent cells through the biological process of cell division. While mitosis controls the propagation of somatic cells, generation of germ cells is controlled by meiosis. The fidelity of mitosis determines the equal division of duplicated chromosomes to the two daughter cells. The first phase of mitosis, that is, nuclear division or karyokinesis is divided into four sub-phases. Prophase marks the initiation of mitosis bringing about chromosome condensation, separation of duplicated centrosomes, and recruitment of some mitotic checkpoint proteins to the kinetochores. Following this, disassembly of the nuclear envelope (NE) marks the entry into metaphase (prometaphase). Subsequently, the release of chromosomes into cytoplasm activates the spindle assembly checkpoint (SAC) at each unattached kinetochore. After microtubule capturing of each chromatid pair at their kinetochores and alignment at the midzone, silencing of the SAC occurs and cell overcomes the “wait anaphase” signal. During anaphase, sister chromatids are completely separated to the two opposite poles of the cell and the invagination of plasma membrane around the spindle midzone becomes visible. Telophase ends with chromosome decondensation and reassembly of the NE around polar chromosomes. Cytokinesis or cytoplasmic division giving rise to two daughter cells follows soon after. Intriguingly, each of these events is sequentially organized in a manner that minimizes any segregational errors. Therefore, defects in the operation of any mitotic event may lead to the generation of chromosomal instability (CIN), a hallmark of cancer (1–5). Having said this, precision and efficiency of the mitotic cell division depends on proper regulation of the expression and function of mitotic proteins. Indeed, most of these proteins show mitotic phase specific activity. This activity is chiefly regulated by post-translational modifications, namely, phosphorylation and ubiquitination, and some other mechanisms (6). Notably, transcription also plays a key role in the maintenance of cell cycle specific protein levels (7). However, little has been summarized about the transcriptional control of the mitotic phenomenon. In this review, we will discuss the role of transcription in mitotic regulation and provide evidence for transcriptional anomalies underlying abnormal mitotic events that lead to CIN and tumorigenesis.

## Mitosis and Aneuploidy

Errors in chromosome partitioning often give rise to aneuploidy. There are several roads that lead to aneuploidy through mitotic errors (4, 8, 9). The first and foremost reason of mitotic aneuploidy is a faulty SAC. The SAC monitors bipolar segregation of duplicated chromosomes during metaphase to anaphase transition (10). Prior to anaphase, sister chromatids remain held together by the cohesin complex (11). At the onset of anaphase, securin gets ubiquitinated by the E3 ubiquitin ligase, anaphase promoting complex/cyclosome (APC/C). This degradation of securin, in turn, makes separase (a protease) free and active. The latter then cleaves cohesin and the chromosomes begin to separate (10, 12). In the presence of any unattached kinetochore or lack of amphitelic attachment SAC is activated. A number of proteins are involved in the tasks executed by the SAC (10). At the molecular level, the mitotic checkpoint complex (MCC) comprised of the Mad and Bub families of proteins, sequesters APC/C adapter protein Cdc20 (13), and APC/C remains inactive until the defects get corrected. After the completion of proper bipolar attachment Cdc20 is ubiquitinated by mitotic ubiquitin carrier protein UbcH10 and gets free from inhibitory MCC (14). Concordant with that, the SAC antagonist protein p31comet binds to the MCC component Mad2 and modulates extraction of Mad2 from MCC. This, in turn, causes disassembly of MCC and blocks further sequestration of Cdc20 (15, 16). Free Cdc20 activates APC/C, which then degrades anaphase inhibitors and cells progress through mitosis. The stepwise functioning of these events depends on the balanced level of the SAC proteins. While mutations in the SAC genes are infrequent in human cancers, their altered expressions are often reported in various cancers and have been associated with defective SAC-mediated aneuploidy (4). Hence, the balanced levels of different SAC proteins are important determinants of SAC behavior. The cell cycle specific transcriptional regulation of SAC proteins might be an elemental reason in maintaining this balance, deregulation of which might be involved in altered levels of the SAC molecules.

In search of other CIN inducing mitotic phenomena, genetic screens have identified cohesion defects as contributors to the onset of aneuploidy (3, 4, 8). Glitches in the machinery monitoring sister chromatid cohesion might promote aneuploidy. Consistent with this, a recent study identified mutations in *STAG2* (which encodes a protein subunit of the cohesion complex) in a number of aneuploid primary tumors and cancer cell lines (17). Also, overexpression of securin and separase, two key regulators of cohesion, is reported to promote aneuploidy and tumorigenesis (18, 19). Chromosome missegregation may also occur in case of merotelic attachment where a single kinetochore attaches to microtubules emanating from both poles of the spindle (20, 21). Several molecular components, for example, Aurora kinase B, kinesin-13 proteins, MCAK, INCENP, Survivin, and Shugoshin are associated in this phenomenon and their overexpression are reported in cancers of various origins (21). A final source of aneuploidy is the prevalence of aberrant centromeres and multipolar mitosis (2–4). Centrosomes provide mitotic spindle poles and concurrently, presence of more than two centrosomes might produce multipolar spindles. Additionally, aberrant chromosome numbers and multipolarity are associated with CIN in various cancers (22). A number of cellular proteins, including Aurora kinase A, Plk1, Chk1, Chk2, Cyclin B1, and Cdk1, regulate centrosome duplication and the abnormal upstream regulation of these proteins is found in various cancers (2).

## Molecular Control of Mitosis: Regulation of Mitotic Proteins

**Figure 1:**
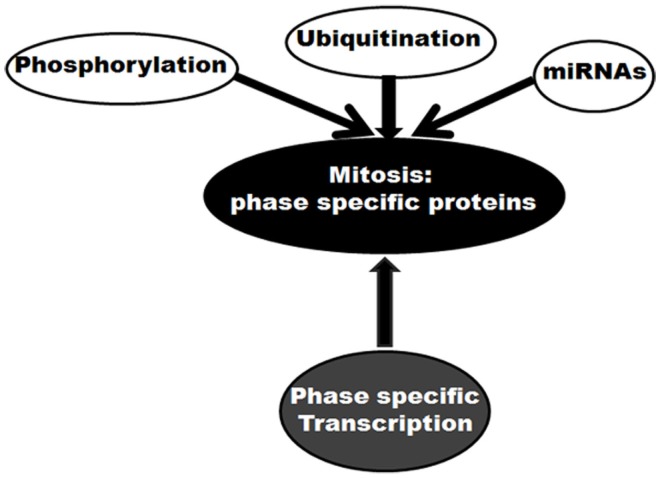
**Molecular control of mitosis: regulation of mitotic proteins**. Different regulatory mechanisms are shown.

Mitosis is a complex event performed by multiple factors with distinct phase specific responsibilities. Regulation at the protein level plays a crucial role in the mitosis specific performances by these factors. These regulations can occur through several routes (Figure 1). First, ubiquitination-mediated protein degradation is believed to be pivotal. The mitotic ubiquitin ligase, APC/C promotes ubiquitination of various protein substrates in a spatial manner (23). By ubiquitinating and consequently targeting mitotic inhibitors for proteasomal degradation, this cellular phenomenon controls mitotic progression in a unidirectional manner. Second, phosphorylation controls functional activities of a number of mitotic proteins in a time-dependent manner. Mitotic cyclin dependent kinase Cdk1, in association with Cyclin A or B, phosphorylates more than 70 substrates involved in various steps of mitosis (24). Some other mitotic kinases like Aurora, Polo, and Nek families also participate in phosphorylation-mediated mitotic regulation (24). As a third mechanism, microRNA (miRNA)-mediated regulation of mitotic proteins is also currently emerging (25–30). In this list of regulatory pathways, the control of expression at the transcription level could be considered as momentous.

## Roads to Chromosomal Instability: Contribution of Mutation Versus Transcription of Mitotic Genes

**Figure 2:**
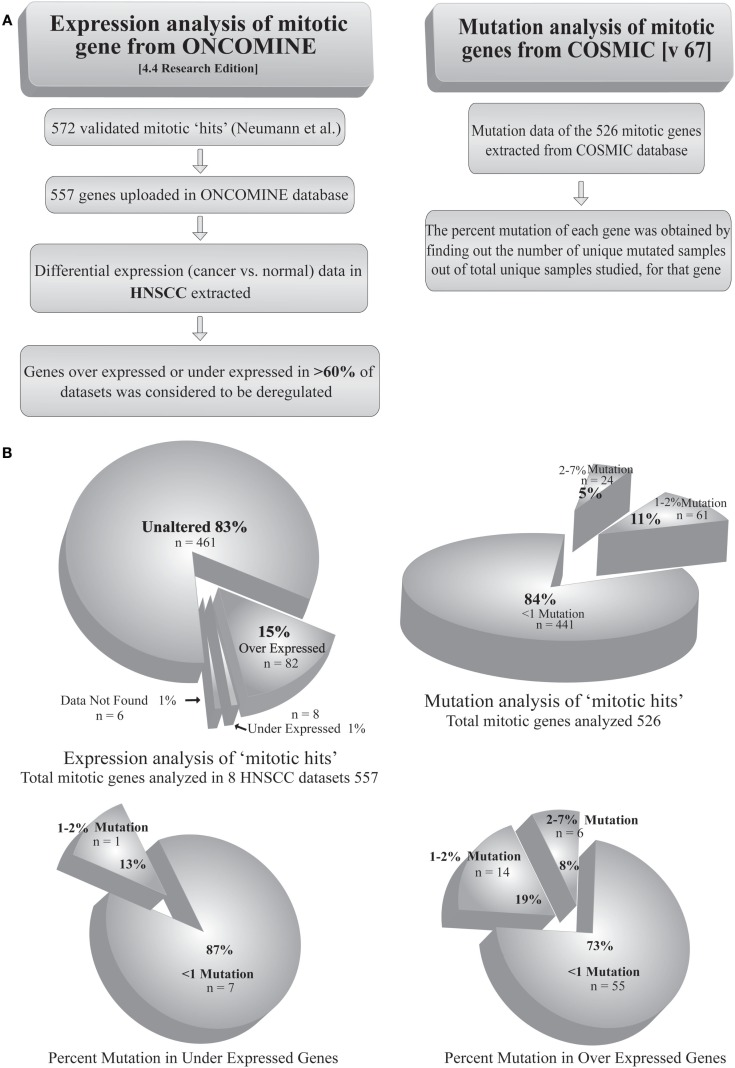
**Analysis of mutation and transcriptional alteration in mitotic genes**. **(A)** Expression analysis of mitotic genes was done using ONCOMINE (4.4 research edition) database and mutation analysis was done using COSMIC (v67) according to the given workflow. **(B)** The two analyses were correlated to obtain the percentage of mutations in overexpressed and downregulated genes.

Most of the tumors are reported to acquire a number of mutations in proto-oncogenes and tumor suppressor genes. Mutation of a gene may alter its product, qualitatively or quantitatively. Extensive search has shown mutations in >1% of candidate genes causally related to oncogenesis, termed as cancer genes (31). Given the fact that mitotic protein levels are pivotal in proper execution of mitosis, the mutational defects can be assumed prime factors in deregulation of mitosis. Simultaneously, a few reports have identified mutations in SAC as well as other mitotic genes in cancers of different origins (32–34). For example, a biallelic germline mutation of the SAC gene *BUB1B* has been diagnosed with mosaic variegated aneuploidy, a rare recessive condition of childhood cancer (35). The genetic alterations, such as gene amplification or depletion, also play a key role in the regulation of many mitotic genes. For example, the genes expressing Aurora-A and Ect2 are amplified in several types of tumors (36–38). Interestingly, despite these reports, mutations directly affecting a mitotic gene are not frequent among cancer types. In an *in silico* approach, we analyzed the mutation status of 526 genes from a list of 572 validated mitotic genes (39) using COSMIC v67 database^1^ (Table S1 in Supplementary Material). The percent mutation for each of these genes was obtained from the percentage of unique mutated samples out of total samples studied. The extracted dataset showed <1% of mutations in 84% (441) of the genes. On the other hand, only 5% of the genes showed mutations in >3% of the samples (Figure 2). In a separate approach, we tried to find out the expression status of validated mitotic hits (39). Using ONCOMINE 4.4 research edition database^2^ cancer versus normal expression patterns were obtained for 557 mitotic genes in 7 head and neck squamous cell carcinoma (HNSCC) datasets (Table S2 in Supplementary Material). Among these, 15% (82) of the genes were found to be overexpressed and 1% was found to be down-regulated in >60% of datasets (Figure 2). To find out whether mutations are responsible for the altered expression of these genes we correlated these two analyses (Table S3 in Supplementary Material). The analysis revealed that 73% of the overexpressed mitotic genes have mutations in <1% of the samples. Only 19% of the overexpressed genes were detected with mutations in 1–2% samples and 8% of the genes were detected with mutations in >3% of the samples. Among the down-regulated genes, 87% of the genes showed mutations in <1% of the samples while 13% of the genes showed mutations in 1–2% of the samples (Figure 2). This data clearly negate the involvement of mutations in regulating the expression of mitotic genes. The probable reasons behind these findings could be (a) mutation in any one of the mitotic genes including SAC regulators may weaken the checkpoint or other mitotic regulations; (b) mutation leading to complete inactivation of any crucial mitosis regulatory gene would be fatal and be eliminated by death of the affected cell(s). For instance, germline deletion of the SAC gene *MAD2* is associated with the loss of pregnancy (40). Indeed, negligible cancer-associated mutations are reported for Aurora kinase B, Cdk1, Cyclin B, Nek2, and Pin1, proteins involved in initial events of mitosis (Table S1 in Supplementary Material) (6). Also, mutations in mitotic checkpoint genes themselves are not found responsible for abnormal checkpoint in cancer cells (8) and infrequently reported for core SAC proteins like Cdc20, Bub3, and Mad2, and SAC-associated proteins like Borealin, Zwint, Hec1, and Aurora kinase B (6).

## Transcriptional Control of Mitotic Genes

**Figure 3:**
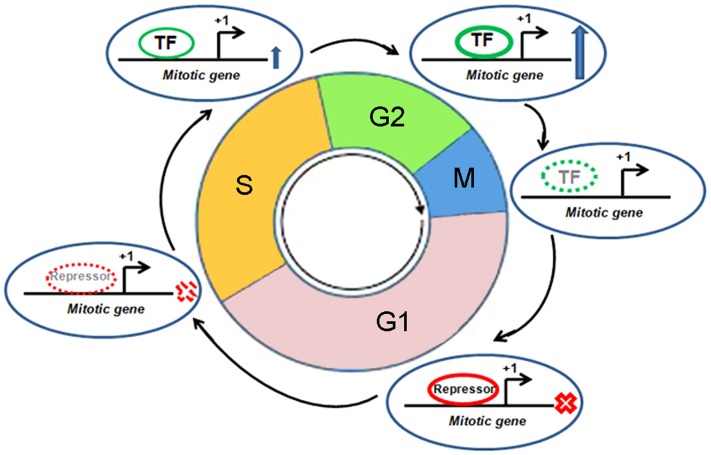
**Cell cycle specific transcriptional regulation of a mitotic gene**. Different cell cycle phases, mitotic gene, transcription start site, transcription factor (TF), and repressor are shown. “X” mark indicates “transcription off” condition and up arrow indicates “transcription on” condition. Dotted appearance indicates gradual reduction of recruitment of TF or repressor.

Mitosis, like any other pathway, is essentially interplay among various protein molecules with tightly regulated phase specific functional activities. A number of mitotic genes show peak level of transcription when the cell passes through the G2 phase (Figure 3) (41). Promoters of these genes remain repressed during G0 and G1 phase. The relief from repression starts at the S-phase and peaks after reaching the G2 phase. The transcription factor, NF-Y is crucial in this timely expression (41). A number of mitotic genes contain two or three CCAAT boxes. These sites are recruited by hetero-trimeric NF-Y in association with histone acetyltransferase p300. This dynamic recruitment brings upon transcriptional activation of mitotic genes at the late phase of cell cycle (42). Two consensus sites, namely, cell cycle dependent element (CDE) and cell cycle genes homology region (CHR), have been extensively described in the global regulation of genes having mitosis specific expression (41). Transcriptional repression remains maintained during G0 and G1 phase through the binding of repressor proteins in CDE and/or CHR elements. The release from repression starts at the S-phase. Following this, activation of these promoters mostly occurs through CCAAT boxes after binding of NF-Y in combination with the co-activator, p300. Promoters of mitotic genes, namely, CCNA (Cyclin A), CCNB1 (Cyclin B1), CCNB2 (Cyclin B2), CDC2/CDK1, CDC25C, CKS1, MKLP1, PLK1, and TOME-1, with well documented mitotic phase specific regulation by CDE/CHR elements, are found to be activated through their CCAAT consensus elements (41). On the other hand, p53 has been associated with the repression of several mitotic genes through CDE/CHR elements (41). A number of mitotic genes, like CDC20, CKS1, CCNB1, CCNB2, and CDC2/CDK1, are repressed by p53 (43–46). However, cell cycle specific repression of some other genes without CDE/CHR has also been documented (41). Toward that, besides CDE/CHR site driven effect, a direct p53 binding element has been identified to regulate Cdc20 expression (47).

Beside this, several other transcription factors have also been reported to control the expression of genes in mitosis specific manner. As, for example, Forkbox M1 (FoxM1) has been identified as a master regulator of mitosis. Laoukili et al. have elegantly shown a transcriptional cluster to be regulated by FoxM1 (48). Another study conducted by Wang et al. also ended up with similar observation for FoxM1 as a master regulator of mitotic genes, like CDC25A, AURKA, AURKB, Survivin, CENPA, CENPB, CKS1, SKP2, and PLK1 (49). Subsequently, Fu et al. have shown that mitotic serine/threonine kinase protein, Polo like kinase 1 (PLK1), a target of FoxM1 itself, interacts with and phosphorylates FoxM1. This phosphorylation, indeed, regulates the transcriptional program driven by FoxM1 (50), thereby indicating a positive feedback loop as a driving force in mitotic transcriptional regulation.

In the last decade, E2F transcription factor family, well-known regulator of S-phase specific trans-regulation, has also been identified in transcriptional control of mitotic genes (51–57). The initial finding of E2F targets from microarray analysis was validated in more than one way and was followed up with identification of target genes involved in chromosome condensation and segregation, SAC functioning, centrosome organization and duplication, and cytokinesis. For instance, core SAC protein Mad2, mitotic ubiquitin carrier protein UbcH10 and PTTG1, a subunit of chromosome cohesion regulator Securin are shown in extensive detail to be G2/M specific E2F targets (58–60). Zhu et al. further showed that recruitment of an activator E2F to the promoter of mitotic cyclin-dependent kinase gene CDC2/CDK1 requires an adjacent CCAAT consensus site pre-occupied by NF-Y. Furthermore, the authors reported that the association of Myb family transcription factor, b-Myb to the promoter of CDC2 and CCNB1 depends on an intact E2F binding site, suggesting a co-operative nature of trans-factor binding in determining mitotic gene activation. Interestingly, b-Myb, itself being an early phase E2F target, links the E2F driven early phase (G1/S) and late phase (G2/M) transcription cascade. Cdc2, Cyclin A2, and Cyclin B1, three important regulators of mitotic entry and progression, were found to be under control of b-Myb-E2F mediated transcription (61).

Human MuvB core complex, comprising of Lin9, Lin37, Lin52, Lin54, and RBBP4, was also identified to regulate transcription of the genes required for the progression into mitosis. Knockdown of the members of this complex led to downregulation of mRNA levels of mitotic proteins including Plk1, Aurora kinase A, Bub1, CENP-E, Lap2, Cyclin A2, Cyclin B1, Cep55, Survivin, and Cdc2 (62, 63). Following this, Sadasivam et al. (64) explains the association and interplay among these master regulators of transcription during the course of cell cycle. They showed that DREAM complex (comprising of *D*P1, *R*b-related protein p130, *E*2F4, *a*nd *M*uvB core complex) functions as a global repressor of mitotic genes during quiescence or G0 phase. Following entry of a cell in G1 phase after quiescence, this DREAM complex dissociates from MuvB core complex. The MuvB core complex then associates with b-Myb and gets recruited to the promoters of late phase mitotic genes (64). Subsequently, MuvB and b-Myb together facilitate the binding of FoxM1 to these promoters during G2 phase to promote the transcription of mitotic proteins like, Cyclin B1, Plk1, Cdc6, Aurora kinase A, and RacGAP1. The cell cycle regulated expression of three other mitotic genes, namely ECT2, MgcRacGAP, and MKLP1, also showed CHR dependent repression throughout G1 phase (65). These genes code for three important regulators of Rho GTPases, critical for mitotic progression, and cytokinesis. The cut homeobox 1 (Cux1) transcription factor coordinately induces the expression of these three genes from S-phase. Moreover, E2F1 was shown to be required in this Cux1 dependent trans-activation process (65).

Besides these well-known consensus elements and master regulators of transcription, some gene specific regulations are also documented in influencing the expression of several mitotic genes. For instance, the transcription of Cdc20 is reported to be regulated by E2F through a new element called Cell cycle *Si*te *R*egulating p55Cdc/*F*izzy transcription (SIRF) (66). Surprisingly, a few mitotic proteins are also identified with transcription regulatory activities. A report showed that WD40 domain containing mitotic checkpoint proteins could act as co-repressors during interphase. The WD40 domain containing SAC proteins, Cdc20 and Bub3, form a complex with histone deacetylases (HDAC1 and HDAC2) during the course of repression (67). On the other hand, we showed that Cdc20, in combination with APC/C and CBP/p300, transcriptionally activates the expression of UbcH10 (68). Furthermore, recruitment of this Cdc20 trans-complex showed dependence on E2F consensus element on the *UBCH10* promoter (60). The mitotic kinase Plk1 was reported to regulate mitotic gene transcription program by phosphorylating FoxM1 (50). Together, these findings clearly indicate a co-ordination of several master regulators of transcription among themselves and with some gene specific co-activators in controlling cell cycle specific expression of mitotic players. This, in turn, points out the importance of transcription in maintenance of mitotic progression.

## Transcriptional Alterations of Mitotic Genes and Association with Cancer

**Figure 4:**
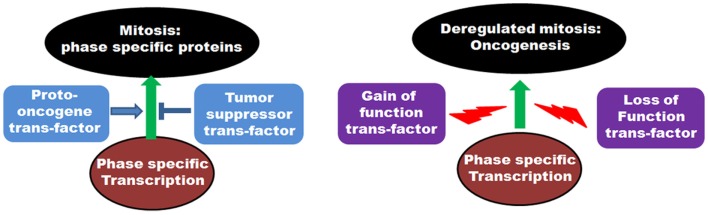
**Role of transcription factors in regulation of mitosis: The left panel shows the involvement of various proto-oncogenic trans-factors as well as tumor suppressor transcription factors in regulation of phase specific expression of mitotic proteins**. The right panel depicts the deregulation of transcription by gain of function mutations of proto-oncogene trans-factors as well as loss of function mutations of tumor suppressor trans-factors and onset of oncogenesis.

On a cellular level, cancer cells are associated with the loss-of-function mutations of tumor suppressors and the gain-of-function mutations of proto-oncogenes. As many of the mitotic genes are transcriptionally regulated by tumor suppressor or proto-oncoprotein trans-factors, the above-mentioned mutational incidences frequently deregulate the transcriptional outcome of the mitotic genes in tumor cells (Figure 4). This, in turn, results in the abnormal execution of mitosis and defects in the chromosomal segregation leading to aneuploidy. Concordant with that, abnormal expressions of many mitotic genes are often associated with the occurrence of oncogenesis.

At the initial stages of mitosis (centrosome maturation, chromosome condensation, NE breakdown, and spindle formation), a number of proteins participate in an orchestrated fashion. Among them, expression of Cyclin B, a Cdk1 activator involved in G2/M progression, has been found to be regulated by the tumor suppressor p53 (69). The direct interaction of p53 to the promoter response element downregulates Cyclin B expression upon DNA damage-mediated checkpoint arrest (69). With alteration of p53 pathway, overexpression of Cyclin B has been shown to contribute to the alteration of SAC and occurrence of CIN in cancer samples (70–72). The Ser–Thr kinase, Plk1 (involved in mitotic initiation in more ways than one) showed elevated mRNA levels in a variety of tumors (73). This protein is transcriptionally coordinated during cell cycle, its level being low during interphase and maximum in mitosis (74). The cell cycle-dependent repression of Plk1 is mediated by Rb pathway. During DNA damage-mediated checkpoint activation, tumor suppressors like p53 and BRCA1 are found to influence levels of Plk1 (74, 75). Correlated with the loss of functional tumor suppressors, transcriptional deregulation of Plk1 is reported in various cancers and associated with CIN and oncogenic transformation (76). Furthermore, tumor suppressors like BRCA1 and Rb are reported to regulate the levels of another mitotic kinase Nek2. In co-ordination with the loss-of-function of tumor suppressors, the overexpression of this protein is associated with CIN and cancer (77–80). Cell cycle specific expression of mitotic Aurora kinases (Aurora kinase A and Aurora kinase B) are CDE-CHR element regulated (81, 82). Oncoproteins like EWS-Fli1 and Myc upregulate expression of aurora family proteins through binding on promoter response elements. On the other hand, tumor suppressors like p53, Brd4 also influence expression of Aurora kinases (83–86). In fact, the altered expression of Aurora kinases are potential markers of cancer progression (87). Regulated expression of kinetochore protein Hec1 is directly related to phosphorylation-mediated inactivation of Rb during the course of cell cycle. Beside this, the CREB family of oncoprotein transcription factors has been shown to upregulate the levels of kinetochore protein Hec1 (88). Disrupted pRb function is associated with transcriptional upregulation of Hec1, which may cause aneuploidy and tumor formation (89, 90).

The initial events of mitosis are followed by chromosomal alignment at the equatorial plane of the cell during metaphase. The amphitelic metaphase alignment precedes SAC release, chromosomal segregation, and entry into anaphase. Consistent with their mitotic roles, a number of core SAC and SAC-associated proteins (Mad1, Mad2, Bub1, Cdc20, UbcH10 to name a few) accumulate gradually through the G2 phase with peak levels at mitosis (13, 68, 91, 92). Different transcription factors and chromatin modifiers regulate cell cycle specific promoter activities of these genes (68, 92, 93). The well-known tumor suppressor p53 is reported to control the transcription of *CDC20* and *BUB1B* (46, 47, 94). Upon genotoxic stress, Cdc20 expression is indirectly suppressed by p53 through p21-dependent mechanism (46). On the other hand, a direct p53 binding element has been identified on the *CDC20* promoter and shown to bring about repression of transcription through chromatin remodeling (47). Similarly, direct recruitment of p53 on the promoter consensus element brings about chromatin remodeling and the repression of Mad1 expression (95). Expression of Mad2 is regulated by E2F in a cell cycle-dependent manner. Rb inactivation leads to aberrant Mad2 expression by deregulating E2F activity and contributes to mitotic defects and aneuploidy (58). The tumor suppressor BRCA1 was also reported to regulate Mad2 expression (96). Cancer-associated defects in these tumor suppressors contribute to the abnormal expression of these proteins and a flawed SAC. Indeed, transcriptional abnormalities including differential promoter methylation of these SAC proteins are potential markers of cancers of various origins (97, 98). Their deregulated expressions are associated with CIN phenotype and incidence of cancer (6).

The final stages of mitosis involve cytokinesis and mitotic exit. Along with mitotic kinases like Aurora, Polo, and related families, some other molecular components also regulate this stage of the cell cycle. Protein regulator of cytokinesis 1 (PRC1) and the guanine nucleotide exchange factor, Ect2, the two major molecules of cytokinesis have been related with cancer-associated altered expressions and CIN (6). In conclusion, we have summarized a number of reports from the ever-growing lists of proto-onco gene as well as tumor suppressor trans-factors in regulation of mitosis and their deregulation in tumor background (Table 1).

**Table 1:** **Role of proto-oncoprotein and tumor suppressor transcription factors in mitosis and involvement in oncogenesis**.

Transcription factor	Mitotic target	Reference
**PROTO-ONCOPROTEINS**		
c-Myc	Aurora kinase A and Aurora kinase B	(83, 99)
c-Myc	Mad2 and BubR1	(100)
c-Myc	Cyclin B1	(101)
Epstein–Barr virus nuclear antigen 2	Mad2, Plk1	(102)
FoxM1	Cyclin B1, CENP-F, Plk1, Nek2, Aurora kinase B, Cyclin	(48, 103–105)
Mutant p53	Cyclin A, Cyclin B1, Cyclin B2, Cdk1	(106)
Mutant p53	Mad1	(107)
EWS-Fli1	Aurora kinase A and Aurora kinase B	(85)
CREB	Hec1	(88)
CREB	Cyclin A	(108)
**TUMOR SUPPRESSORS**		
BRCA1	Mad2	(96)
BRCA1	BubR1, Hec1, Stk6, Nek2, Securin, Prc1, Plk, Knls2, Cdc2, and Cdc20	(78)
Rb	Mad2	(58, 109)
Rb	Hec1	(90)
Rb	UbcH10	(60)
p53	Cdc20	(46, 47)
p53	Mad1	(95)
p53	Aurora kinase A, Plk2, Lats2	(110)
p53	Cyclin A1	(111)
p53	Cyclin B	(45)
p53	Emi1	(112)
pVHL	Mad2	(113)

## Conclusion

The role of transcriptional regulatory pathways behind the incidence of tumorigenesis remains an enigma. For a number of key cell cycle regulators, the transcriptional control represents an evolutionarily conserved mechanism to precisely maintain their abundance, working in conjunction with miRNA mediated silencing, translational control, and ubiquitin-mediated degradation (23, 26, 114, 115). Among these cell cycle regulators, a defined set of factors stringently control mitotic entry, progression, and exit. The interplay among these factors is naturally adjusted by their abundance. Abnormality in this abundance is associated with the occurrence of aneuploidy, a hallmark of cancer (2, 8, 116). In this review, we have discussed the maintenance of protein levels of the mitotic players whose transcription is regulated in a cell cycle specific manner. We further discussed the deregulation of their transcriptional control, working in concert with cancer onset. In this regard, mutations in various tumor suppressors and proto-oncogenes acting as co-factors of transcription are found to disharmonize the relative protein levels, rather than mutations in the mitotic genes themselves. Besides this, a few mitotic genes are reported to participate in transcriptional control. Furthermore, the list of transcripts whose transcription is affected by certain cell cycle or developmental transitions is being expanded owing to new genome-wide approaches. Answer to many open questions regarding the interplay between transcriptional regulation and mitotic progression will make an important contribution to the understanding of cell cycle control. This, in turn, will help to dissect the involvement of cell cycle progression in the onset of tumorigenesis.

## Conflict of Interest Statement

The authors declare that the research was conducted in the absence of any commercial or financial relationships that could be construed as a potential conflict of interest.

## Supplementary Material

The Supplementary Material for this article can be found online at http://journal.frontiersin.org/article/10.3389/fendo.2015.00060

## Footnotes

^1^cancer.sanger.ac.uk/cancergenome/projects/cosmic/

^2^http://www.oncomine.org/resource/login.html
